# Qualification of New Methods for Measuring In Situ Rheology of Non-Newtonian Fluids in Porous Media

**DOI:** 10.3390/polym12020452

**Published:** 2020-02-14

**Authors:** Jørgen Gausdal Jacobsen, Behruz Shaker Shiran, Tormod Skauge, Kenneth Stuart Sorbie, Arne Skauge

**Affiliations:** 1 Department of Chemistry, University of Bergen, Allegaten 41, N-5020 Bergen, Norway; 2 Norce, Norwegian Research Centre AS, Allegaten 41, N-5020 Bergen, Norway; besh@norceresearch.no; 3 Energy Research Norway, N-5020 Bergen, Norway; tormod.skauge@energyresearch.no (T.S.); k.sorbie@hw.ac.uk (K.S.S.); 4 Institute of Petroleum Engineering, Heriot Watt University, Edinburgh EH14 4AS, UK

**Keywords:** EOR, polymer flooding, in situ rheology, non-Newtonian flow, polymer memory effect

## Abstract

Pressure drop (Δ*P*) versus volumetric injection rate (*Q*) data from linear core floods have typically been used to measure in situ rheology of non-Newtonian fluids in porous media. However, linear flow is characterized by steady-state conditions, in contrast to radial flow where both pressure and shear-forces have non-linear gradients. In this paper, we qualify recently developed methods for measuring in situ rheology in radial flow experiments, and then quantitatively investigate the robustness of these methods against pressure measurement error. Application of the new methods to experimental data also enabled accurate investigation of memory and rate effects during polymer flow through porous media. A radial polymer flow experiment using partially hydrolyzed polyacrylamide (HPAM) was performed on a Bentheimer sandstone disc where pressure ports distributed between a central injector and the perimeter production line enabled a detailed analysis of pressure variation with radial distance. It has been suggested that the observed shear-thinning behavior of HPAM solutions at low flux in porous media could be an experimental artifact due to the use of insufficiently accurate pressure transducers. Consequently, a generic simulation study was conducted where the level of pressure measurement error on in situ polymer rheology was quantitatively investigated. Results clearly demonstrate the robustness of the history match methods to pressure measurement error typical for radial flow experiments, where negligible deviations from the reference rheology was observed. It was not until the error level was increased to five-fold of typical conditions that significant deviation from the reference rheology emerged. Based on results from pore network modelling, Chauveteau (1981) demonstrated that polymer flow in porous media may at some rate be influenced by the prior history. In this paper, polymer memory effects could be evaluated at the Darcy scale by history matching the pressure drop between individual pressure ports and the producer as a function of injection rate (conventional method). Since the number of successive contraction events increases with radial distance, the polymer has a different pre-history at the various pressure ports. Rheology curves obtained from history matching the radial flow experiment were overlapping, which shows that there is no influence of geometry on in-situ rheology for the particular HPAM polymer investigated. In addition, the onset of shear-thickening was independent of volumetric injection rate in radial flow.

## 1. Introduction

Polymer flooding is a mature enhanced oil recovery (EOR) technique that has received increased attention during recent years. Water-soluble polymers are added to the injection brine to increase effective viscosity of the injected solution, thus changing the mobility ratio and thereby improving sweep efficiency. The synthetic polymer partially hydrolyzed polyacrylamide (HPAM) is by far the most frequently used polymer for oil recovery purposes [1] and is therefore the focus of this paper. While biopolymers are purely viscous in solution and thus only exhibit Newtonian and shear-thinning behavior, elastic polymers such as HPAM additionally exhibit shear-thickening behavior in flow through porous media.

Accurate measurements of polymer in situ rheology is crucial for obtaining reliable estimates of the mobility ratio between the displacing polymer solution and the displaced oil. Due to the time-consuming nature of in situ measurements, extensive efforts have been made to relate bulk and in situ rheology. Despite numerous attempts, no universally accepted analytical or numerical model exist [2]. Consequently, polymer in situ rheology is estimated from polymer flow experiments in porous media. Generally, these experiments have been performed on linear core plugs [3,4,5,6,7]. However, results from recent years indicate that linear and radial polymer flow are inherently different [2,8]. In addition, it has been observed that presence of residual oil significantly reduces polymer in situ effective viscosity compared to single-phase flow [2]. Based on these results, the radial polymer flow experiment that was history matched in this paper was performed in radial flow geometry in the presence of residual oil since these conditions best mimic the polymer flow out from the injector in oil reservoirs.

Until recently, in situ polymer rheology was mainly calculated from the pressure drop (Δ*P*) along a linear core as a function of volumetric injection rate (*Q*). Using this Δ*P* versus *Q* data directly, the behavior of the in situ ‘effective viscosity’ could be calculated using Darcy’s law. Consequently, a single effective viscosity value could be obtained for each volumetric injection rate. Unlike the case in linear flow, the velocity is decreasing with distance from the injector in a radial flow system. Thus, internal pressures in radial flow provide a much richer description of the local pressure response from both the shear-thickening regime in the near-wellbore region and the shear-thinning regime further from the injection well. Even though internal pressure ports are also used in linear core floods, they are unable to provide any additional detail from the polymer pressure response due to the constant velocity field in linear flow. Extrapolation of internal pressures may be performed to obtain the polymer entrance pressure drop in linear flow, which is correlated with the degree of mechanical degradation [9]. However, recent results show that the degree of mechanical degradation is significantly reduced in radial compared to linear flow [8]. Therefore, it is the authors’ view that linear flow experiments should not be used as basis for determining the degree of mechanical degradation in radial flow.

A common aspect of all polymer flow experiments, regardless of flow geometry, arises from uncertainties originating from pressure measurement error, which may significantly influence the history match results. Moreover, it has been suggested that the observed shear-thinning behavior of HPAM solutions may be an experimental artifact due to the use of insufficiently accurate pressure transducers [7]. A generic simulation study was therefore performed to establish the sensitivity of both history match methods to pressure measurement error. Initially, a reference (base case) rheology curve was constructed and used as input for radial polymer flow simulations to create a reference dataset. This reference dataset was then randomly ‘contaminated’ with different degrees of pressure measurement error. Rheology curves used to history match datasets with different levels of pressure measurement error were then compared to the reference rheology curve. Consequently, the effect of pressure measurement error on the resulting polymer rheology function could be quantitatively evaluated.

Due to the rapidly changing velocity field in radial flow (radial velocity decreases as 1/*r*), different flow regimes may exist within the propagation path between injector and producer for elastic polymers. When the volumetric injection rate and resulting flow velocities are below a certain threshold value, the polymer will have sufficient time to relax completely between each contraction during the entire propagation distance and the fluid can then be treated as an inelastic fluid for modeling purposes. However, at velocities exceeding this threshold value, the polymer is unable to fully relax between contractions and the elastic nature of the polymer must be considered. Chauveteau (1981) [10] showed that for elastic fluids, a memory effect is observed where the polymer rheology is dependent on the number of prior contraction events that the fluid experiences. These experimental observations have also been reproduced by numerical simulation [11], where it was demonstrated that porous media properties such as; aspect ratio, coordination number, and tortuosity, significantly influence polymer rheology.

The use of internal pressure ports in the radial flow experiment, where the number of contraction events increase with distance from injection well, enabled an investigation at the Darcy scale into the memory effects of the elastic HPAM polymer considered in this paper.

## 2. Materials and Methods

### 2.1. Rock Material

The radial flow experiment history matched in this paper was performed on a circular Bentheimer sandstone disc. Based on XRD measurements, Bentheimer consists predominantly of quartz (90.6%) with some feldspar (4.6%), mica (3.2%), and trace amounts of minerals such as siderite, calcite, and pyrite (1.6%). Mercury intrusion tests performed on the Bentheimer outcrop rock showed a uniform and relatively narrow pore size distribution with median pore throat size of approximately 14.7 µm [12]. Dispersion tests were also performed, where the flow behavior of the experimental brine and a tracer brine confirmed homogeneous flow conditions. Table 1 shows the properties of the Bentheimer sandstone disc used in this study.

**Figure 1:**
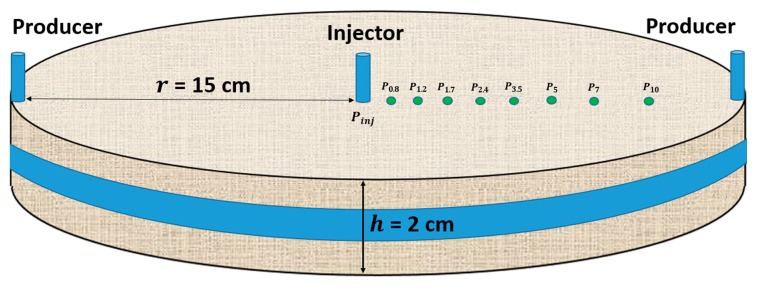
Schematic representation of the circular Bentheimer disc showing the distribution of internal pressure ports mounted between injection well and production line.

The sample was prepared according to a method previously described in the literature for circular Bentheimer discs with internal pressure ports [2,8,13]. The rock sample preparation was completed by ageing with a heavy crude oil of 7000 cP, followed by flooding with brine to residual oil saturation of 0.34. Pressure ports were located in the injection well and production line and at radii of, *r* = 0.8, 1.2, 1.7, 2.4, 3.5, 5, 7, and 10 cm (Figure 1). As evident from the illustration, the entire rim of the radial disc constitutes the production line.

Fuji FCX series pressure transducers were used to measure the absolute pressure for individual pressure ports. These pressure sensors have a pressure range of 0–5 bara with a resolution within ±0.2% of the preset maximum pressure value. Differential pressures between individual pressure ports and production line were then calculated from the measured absolute pressures. In radial flow experiments with internal pressure ports, measurement error originate from both pressure measurement noise in pressure tranducers and uncertainties from the manual placement of internal pressure ports. Consequently, the effective (or cumulative) pressure measurement error was estimated at approximately ±1% of the preset maximum value for the radial polymer flow experiment conducted in this paper.

### 2.2. Fluids

The brine used in this study was of relatively low salinity and did not contain any divalent ions. Total dissolved salt content was equal to 7000 ppm, with composition given by 6000 mg/L NaCl and 1000 mg/L NaHCO_3_. Brine viscosity was 1.02 cP at the experimental temperature of 22 °C. The 1000-ppm polymer solution reported in this paper was prepared by diluting a 5000 ppm mother solution (Flopaam 3330S, 8 MDa, 30% hydrolysis, from SNF Floerger, Andrèzieux-Bouthèon, France) in brine according to the API method (RP 63, 1900, American Petroleum Institute, Washington, D. C., USA). According to the classification of polymer phase categories [14], the polymer solution was within the semi-dilute regime.

The relatively low molecular weight polymer used in this study was chosen to reduce the amount of mechanical degradation and to minimize the residual resistance factor (RRF). In addition, the rather low polydispersity of the HPAM sample was expected to ensure low mechanical degradation compared to a wider distribution [7].

Shear viscosity of the polymer solution (11.5 cP at 10 s^−1^) was measured at 22 °C using a cone-plate geometry on a Malvern Kinexus Pro rheometer. Shear viscosity was measured for shear rates in the interval 0.1 to 1000 s^−1^ using a 50 mm titanium spindle with a 2° inclination. The low density of the spindle allowed for accurate measurements at low torque values. The plate was 65 mm in diameter and the gap set to 70 µm at the tip. The solution was viscous dominated. Effluent viscosity measurements showed negligible deviation from injected viscosity, indicating that mechanical degradation did not occur during the radial polymer flow experiment.

### 2.3. Disc Flooding Procedure

Firstly, brine was injected at ten different flow rates (0.05–2 mL/min) to determine effective permeability to brine in presence of residual oil. Effective permeability to brine ($k_{b,init}$) was obtained from Darcy’s law for radial flow

(1) $$k_{b,init}=-\frac{\mu Q}{2\pi h\Delta P}ln\frac{r}{r_{e}}$$

where μ is brine viscosity, Q is volumetric injection rate, h is disc thickness, ΔP is the pressure drop between a specified pressure port at radius r and the producer at $r_{e}$.

The 1000-ppm polymer solution was then injected at low flow rate (0.1 mL/min) for at least two pore volumes to ensure that polymer retention was completely satisfied and to achieve stable pressure conditions. Then, the polymer solution was injected at 2 mL/min and the injection rate was reduced and measured in a stepwise manner (10 rate steps) until the stabilized pressure had been recorded for the lowest rate of 0.05 mL/min.

Apparent viscosity of polymer solutions flowing in porous media is represented by the resistance factor (RF), defined as

(2) $$RF=\frac{\Delta P_{P}}{\Delta P_{b,init}}$$

where $\Delta P_{P}$ is the pressure drop during polymer flow and $\Delta P_{b,init}$ is the pressure drop during brine flow before polymer was introduced to the porous media.

Following the 1000-ppm HPAM flood, tapering was performed to minimize residual resistance factor [15], where 700, 400, and 100 ppm polymer solutions were injected sequentially. Lastly, the final brine flood was injected at 10 different flow rates (0.05–2 mL/min) to determine final permeability to brine and to calculate the RRF, defined as

(3) $$RRF=\frac{k_{b,init}}{k_{b,final}}$$

where $k_{b,init}$ is effective permeability to brine before introduction of polymer to the porous media and $k_{b,final}$ is effective permeability to brine after polymer tapering.

### 2.4. Simulation

The simulation model used for the generic simulation study and for history matching the radial polymer flow experiment was constructed in the STARS simulator (Computer Modeling Group, Calgary, Alberta, Canada). Concentric grid blocks of uniform length delineated the radial grid with radius of 15 cm between injector and producer. After sensitivity analysis, grid block length was optimally chosen to be Δ*r* = 0.1 cm, resulting in a radial model with 150 concentric rings.

The rheology curves constructed in this paper were generated using an extended version of the Carreau model [16] that incorporates the Newtonian, shear-thinning, and shear-thickening behavior of elastic polymers, as

(4) $$\mu_{app}=\;\mu_{\infty }+\frac{\mu_{0}-\mu_{\infty }}{{\left(1+{\left(\lambda_{1}u\right)}^{2}\right)}^{\frac{1-n_{1}}{2}}}+\;\mu_{max}[1-\exp\left(-\left(\lambda_{2}u{)}^{n_{2}-1}\right)\right]\;$$

where $\mu_{app}$ is polymer apparent viscosity, $\mu_{\infty }$ and $\mu_{0}$ are limiting Newtonian viscosities at high and low shear limits, respectively, λ and n are empirical polymer constants, u is the superficial velocity of the polymer in porous media and $\mu_{max}$ is the shear-thickening plateau viscosity.

### 2.5. History Match Methods

To model radial polymer flow, Darcy’s law may be applied as

(5) $$\Delta P=-\frac{\mu_{app}Q}{2\pi hk_{b,final}}ln\frac{r}{r_{e}}$$

where ΔP is the pressure drop between a specified pressure port at radius r and the producer at $r_{e}$, $\mu_{app}$ is polymer apparent viscosity, Q is volumetric injection rate, h is disc thickness and $k_{b,final}$ is effective permeability to brine after polymer flow.

Permeability obtained from the final brine flood was used as input during history matching of the polymer flood in order to obtain the flow-dependent rheology behavior of the HPAM solution. Thus, we assumed that effective permeability to brine (after polymer flow) and to polymer were equal. This assumption was justified since X-ray imaging showed no additional oil mobilization during polymer flooding beyond the water flood residual oil saturation.

Using Darcy’s law for radial flow and assuming constant brine permeability, the differential pressure during polymer flow may be history matched as a function of either volumetric injection rate (conventional method [13], denoted dP(*Q*)) or radial distance (new method [2,8], dP(*r*)):

- dP(*Q*): Using the conventional method, injection bottom-hole pressure (BHP) is history matched as function of volumetric injection rate, yielding a single polymer in situ rheology curve. Due to the experimental set up using internal pressure ports distributed between injector and producer, differential pressure between each pressure port and producer may also be history matched as a function of injection rate, yielding an individual in situ rheology curve for each pressure port. While the rheology curve obtained from history matching injection BHP spans the entire velocity interval of the polymer rheology from injector to producer, differential pressure between internal pressure ports span decreasing velocity intervals of the complete rheology curve as we move towards the producer.
- dP(*r*): Using the new method, differential pressure is history matched as a function of radial distance, yielding an individual rheology curve for each volumetric injection rate. Here, rheology curves obtained from each injection rate span different velocity intervals.

Throughout the remainder of this paper, dP(*Q*) and dP(*r*) are referred to as the single port match method (SPMM) and the disc match method (DMM), respectively.

### 2.6. Pretreatment of Polymer Pressure Response: Decoupling Polymer In Situ Rheology

Polymer pressure response from radial flow experiments does not exclusively contain contributions from the in situ rheology. If not accounted for, experimental conditions such as non-uniform residual oil saturation and pseudo-skin effects may distort the true functional relationship of the polymer rheology. However, since these conditions also were present during brine flow, and since X-ray imaging showed no additional displacement of oil by polymer, they can be incorporated into the permeability and effectively be decoupled from the polymer rheology.

Effective permeability to brine across the entire disc (obtained from history matching injection BHP as a function of injection rate during brine flow) was used to history match the polymer flow experiment. However, since the permeability across the entire disc deviated from the local permeabilities between individual pressure ports and producer, correction factors were calculated and internal pressures for the polymer flow were adjusted. Using this pretreatment technique, all experimental factors such as non-uniform residual oil saturation and pseudo-skin effects, which were also present during brine flow, were effectively decoupled from the polymer pressure response. Thus, the contribution of polymer in situ rheology on the radial pressure response could be effectively isolated. The calculated correction factors were between 0.5 – 1.3, and were monotonically decreasing with radial distance.

### 2.7. Pressure Measurement Error Analysis

**Figure 2:**
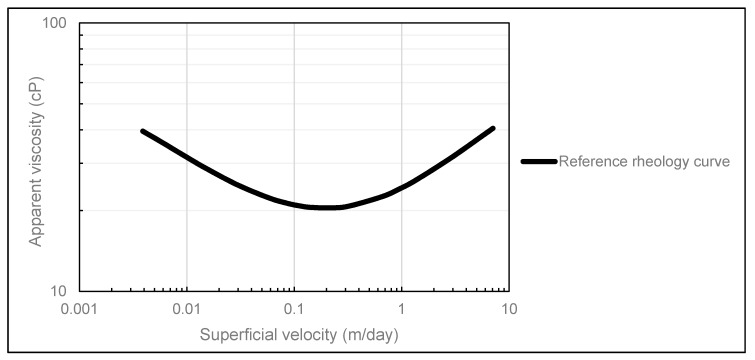
Reference (base case) rheology curve used for pressure measurement error analysis.

To quantify the effect of pressure measurement error on the obtained polymer in situ rheology, the parameter values summarized in Table 2 were used in conjunction with the extended Carraeu model, i.e., Equation (4), to create a reference (base case) rheology curve (Figure 2). This synthetic base case rheology curve is essentially our ‘truth’ case if the data were perfect.

The reference rheology curve was then used as input in our radial polymer flow simulations in STARS to generate a reference dataset. In accordance with the radial polymer flow experiment that was history matched in this paper, the reference rheology curve was used in simulation runs with the same 10 injection rates within the rate interval of 0.05–2 mL/min.

### 2.8. Automatic History Match Tool

To prevent any pre-bias from affecting the results of the error analysis, the automatic history match tool CMOST by Computer Modeling Group (Calgary, Alberta, Canada) was chosen to perform the history match operations. To evaluate the convergence ability of CMOST, the reference dataset was history matched using the particle swarm optimization (PSO) engine. The parameter intervals in Table 3 were selected for all automatic history match operations during the error analysis. These are chosen to give a wide parameter space containing the ‘correct’ value for the base case in situ rheology curve.

History match results using the PSO engine showed good convergence in most situations. However, in some cases, the engine experienced difficulties converging towards acceptable global minimum values. In these cases, the more robust, although more time consuming engine, Bayesian Markov Chain Monte Carlo (MCMC), was used. During the evaluation of CMOST, where the reference dataset was history matched, average history match error of 1% was set as the convergence criterion. In this work, the average percentage history match error is defined as

(6) $$\frac{1}{n}\overset{n}{\underset{1}{\sum }}\left|\frac{HM-R}{R}\right|\times 100$$

where n is the number of pressure ports, HM is the differential pressure obtained by CMOST, and R is the corresponding reference differential pressure.

**Figure 3:**
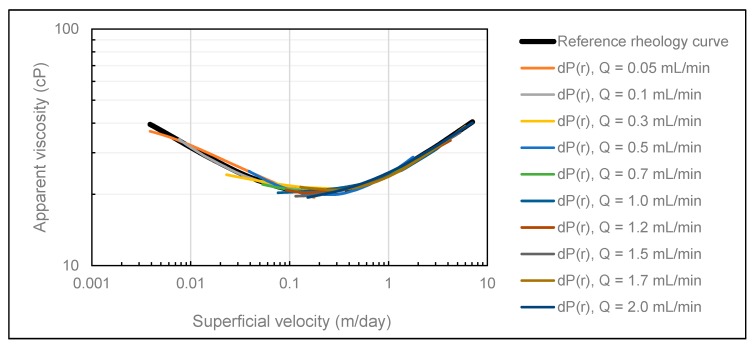
Comparison of the reference rheology curve (black) and rheology curves obtained in CMOST when the reference dataset was history matched using the DMM.

**Figure 4:**
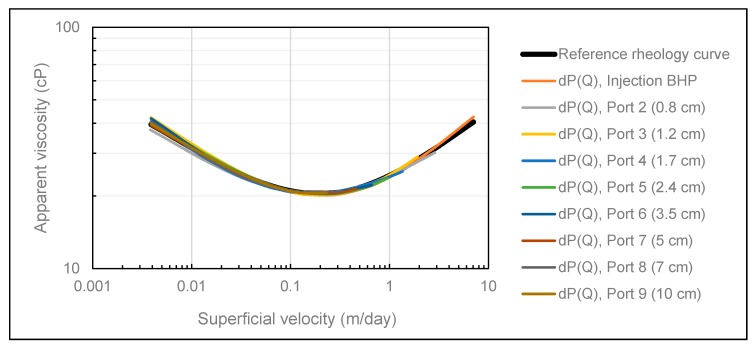
Comparison of the reference rheology curve (black) and rheology curves obtained in CMOST when the reference dataset was history matched using the SPMM.

The history match results obtained using both methods (Figure 3 and Figure 4) confirm the convergence ability of CMOST, and we can conclude that it is an appropriate history match tool for the purpose of this analysis.

To investigate the robustness of both history match methods, pressure measurement errors typical for in-house radial flow experiments were randomly added or subtracted from the reference dataset and this contaminated dataset was then history matched. In accordance with the effective pressure measurement error related to the experimental setup in the radial polymer flow experiment, the dataset was randomly contaminated with ±1% of the preset maximum pressure. The maximum pressure was set equal to the reference injection BHP response for each individual injection rate. Thereafter, the error was increased in a stepwise manner until the threshold level was identified above which the in situ rheology pressure contribution was lost in pressure measurement error.

## 3. Results and Discussion

### 3.1. Pressure Measurement Error Analysis

**Figure 5:**
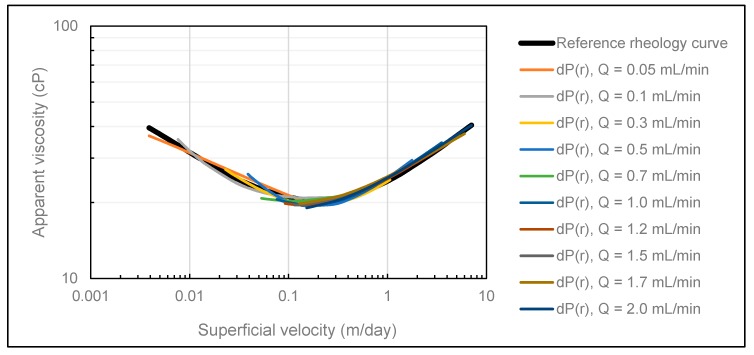
Comparison of the reference rheology curve (black) and rheology curves obtained in CMOST after influence of typical pressure measurement error using the DMM.

**Figure 6:**
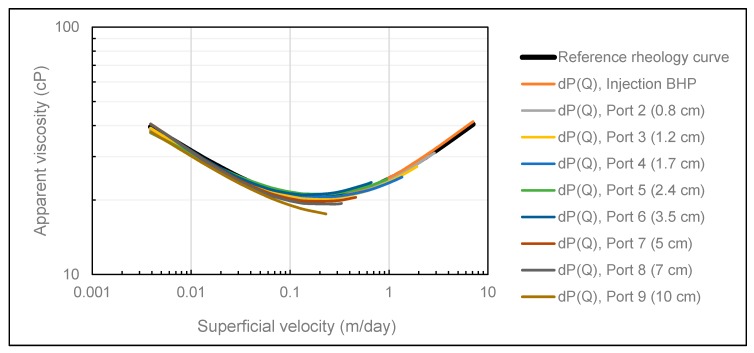
Comparison of the reference rheology curve (black) and rheology curves obtained in CMOST after influence of typical pressure measurement error using the SPMM.

In typical experimental error conditions (1% uncertainty of maximum preset pressure), rheology curves using both history match methods showed negligible deviations from the reference rheology curve (Figure 5 and Figure 6).

These results clearly demonstrate the robustness of both methods under these experimentally realistic error conditions. As expected, we observe that deviation from the reference rheology increase with radial distance, which is readily evident from Figure 6. Based on these results, pressure measurement error typical for in-house radial flow experiments should not be adequate to distort the obtained rheology curves.

To identify the threshold error level above which the polymer in situ pressure response was lost, the error was increased in a stepwise manner until significant deviation from the reference curve was observed. This error level was determined at 5% of maximum preset pressure, which was five times the typical error level. Thus, radial polymer flow experiments should be performed in an experimental setup where effective pressure measurement error is well below 5% of the maximum preset pressure.

### 3.2. History Match of Radial Flow Experiment

The polymer memory effect of HPAM solutions was elegantly demonstrated by Chauveteau (1981) [10] in a series of pore scale experiments in glass models. In said paper, the in situ rheology of HPAM was shown to depend on the number of prior contraction events experienced by the fluid, i.e., on the polymer history. This result was later reproduced by numerical simulation [11], where the polymer memory effect was shown to depend on polymer properties such as molecular weight and porous media properties such as contraction aspect ratio and tortuosity.

**Figure 7:**
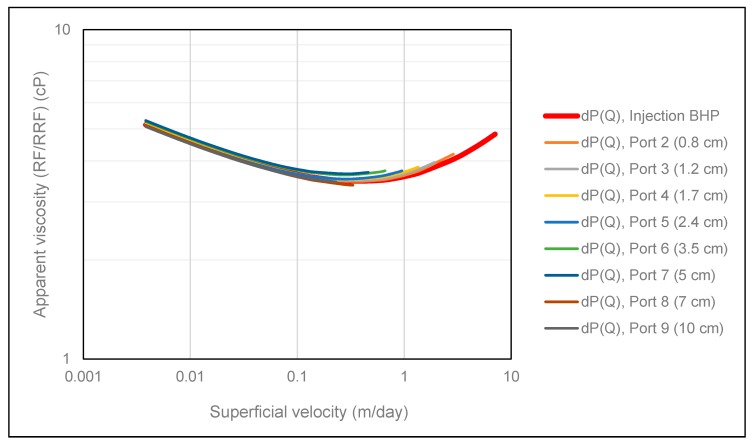
Polymer in situ rheology obtained using the SPMM.

To investigate potential polymer memory effects at the Darcy scale of the elastic HPAM polymer used in this study, differential pressure between each pressure port and producer was history matched as a function of volumetric injection rate. Since the number of contraction events increase with radial distance, the polymer had a different pre-history at different locations in the porous medium. The results from applying this approach are shown in Figure 7, where we observe that all polymer rheology curves are overlapping.

Firstly, this is a strong indication that the pretreatment technique applied to the experimental data was successful in isolating the pressure contribution from the polymer in-situ rheology. However, in circumstances where the polymer is subjected to mechanical degradation, we expect rheology curves obtained from internal pressure ports to deviate from the rheology curve obtained from injection BHP since mechanical degradation mainly occurs in the near wellbore region in radial flow [9]. Thus, injection BHP contains pressure contributions from both degraded and undegraded polymer, in contrast to internal pressures where only pressure response from degraded polymer is recorded. Consequently, we expect the polymer rheology curve obtained from injection BHP to be shifted vertically upwards compared to the rheology curves obtained from internal pressures in circumstances where the polymer is subjected to mechanical degradation in radial flow.

Secondly, since all rheology curves were overlapping, even though they had a different pre-history, no significant memory effects could be observed at the Darcy scale for the polymer solution investigated in this study. Since Bentheimer sandstone is a relatively homogeneous rock, the micro-scale memory effects are most likely averaged out when viewed from a Darcy scale perspective. In accordance with the results demonstrated by Zamani et al. [11], we suggest that memory effects might be observable at the Darcy scale with increasing molecular weight of the polymer and with increasing heterogeneity of the porous medium.

**Figure 8:**
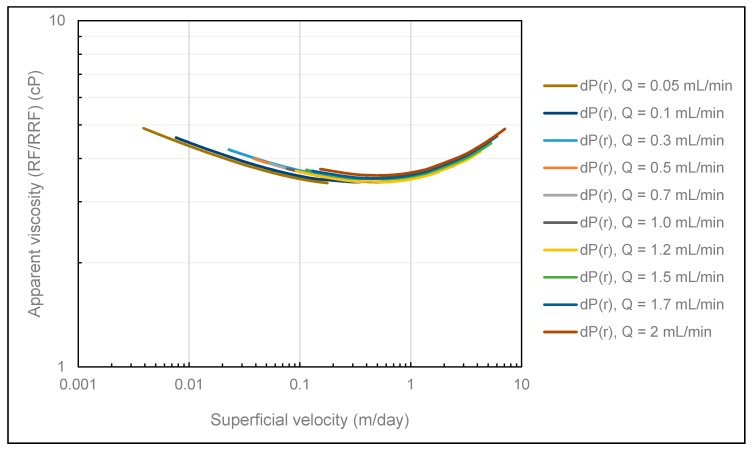
Polymer in situ rheology obtained using the DMM.

Figure 8 shows the obtained polymer in situ rheology when history matching differential pressures using the DMM. Results show no rate effects in that all rheology curves are overlapping. Consequently, the onset of shear-thickening is independent of volumetric injection rate in radial flow. However, in cases where the polymer is subjected to mechanical degradation, rate effects are expected because the amount of mechanical degradation generally increases with volumetric injection rate. In these cases, we do not expect the obtained polymer rheology curves to be overlapping.

**Figure 9:**
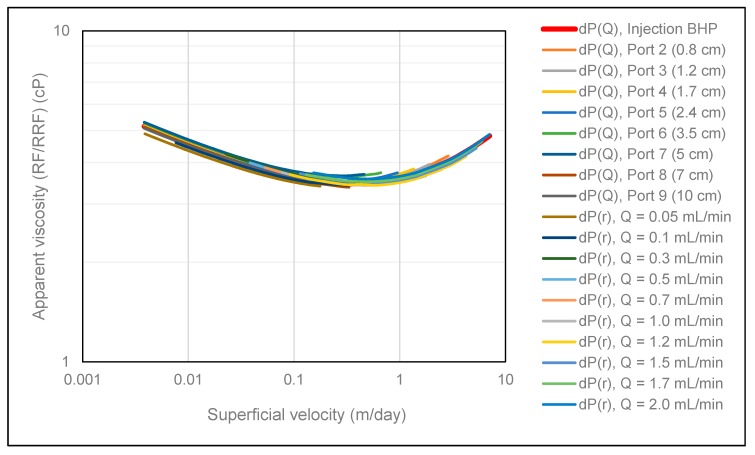
Summary of polymer in situ rheology obtained from both history match methods.

To investigate the consistency between the two different history match methods and their accuracy, all rheology curves obtained from both history match methods are collectively shown in Figure 9. Very good consistency is observed between all curves in Figure 9 in that both history match methods provide overlapping in situ rheology curves. Based on the strong consistency observed, we can conclude that both history match methods are very strong tools for determining polymer in situ rheology in radial flow systems.

## 4. Conclusions

In this paper, two novel methods for measuring in situ polymer rheology in radial flow systems were evaluated. Results from the generic simulation study demonstrated the high accuracy and robustness of both history match methods to pressure measurement error. Here, an upper error level of 5% of maximum preset pressure was identified below which accurate estimates of polymer rheology could be made. Thus, the assertion of shear-thinning behavior of HPAM solutions at low flux in porous media being an experimental artifact due to insufficiently accurate pressure transducers is shown to be unlikely.

Memory effects in radial polymer flow were investigated and were not observable at the Darcy scale in Bentheimer sandstone for the HPAM polymer investigated in this study. However, the authors appreciate that memory effects might be observable at the Darcy scale for higher molecular weight polymers and in more heterogeneous porous media.

No rate effects were observed in that the onset of shear-thickening was observed to be independent of rate in radial flow for the mechanically undegraded polymer in this study.

Finally, polymer in situ rheology obtained from each history match method were compared and showed very consistent results. Thus, both methods evaluated in this paper proved to be robust tools for measuring in situ rheology of non-Newtonian fluids.

## Figures and Tables

**Table 1:** Disc properties

Radius	r	cm	15.0
Injection well radius	$r_{w}$	cm	0.325
Thickness	h	cm	2.00
Pore volume	$V_{p}$	mL	350.0
Porosity	Φ	-	0.25
Permeability	K	mD	2200

**Table 2:** Carreau parameters used to create the reference (base case) rheology curve

$\mu_{\infty }\;(\mathrm{cP})$	$\mu_{0}\;(\mathrm{cP})$	$\mu_{max}\;(\mathrm{cP})$	$\lambda_{1}\;(\mathrm{day}/m)$	$\lambda_{2}\;(\mathrm{day}/m)$	$n_{1}$	$n_{2}$
1	50	75	$5\cdot 10^{7}$	$5\cdot 10^{3}$	0.7	1.5

**Table 3:** Carreau parameter intervals used for automatic history match operations in CMOST

$\mu_{\infty }\;(\mathrm{cP})$	$\mu_{0}\;(\mathrm{cP})$	$\mu_{max}\;(\mathrm{cP})$	$\lambda_{1}\;(\mathrm{day}/m)$	$\lambda_{2}\;(\mathrm{day}/m)$	$n_{1}$	$n_{2}$
1	1–100	1–100	$10^{4}$–$10^{8}$	1–$10^{4}$	0.3–1	1.3–2
